# Endophthalmitis caused by *Abiotrophia defectiva* with initial presentation as retinal vasculitis: a case report

**DOI:** 10.1186/s13256-025-05358-0

**Published:** 2025-07-11

**Authors:** Xiaojie Lu, Bei Liu, Tiemei Yie, Weiwei Wang

**Affiliations:** Shaanxi Eye Hospital, Xi’an People’s Hospital (Xi’an Fourth Hospital), Affiliated People’s Hospital, Northwest University, Xi’an, 710004 Shaanxi China

**Keywords:** *Abiotrophia defectiva*, Endophthalmitis, Retinal vasculitis, Glaucoma, Case report

## Abstract

**Background:**

*Abiotrophia defectiva* is primarily known for its association with endocarditis rather than intraocular infections. We reported a case of endophthalmitis caused by *Abiotrophia defectiva*, presenting as retinal vasculitis in its early stages, a phenomenon rarely documented in literature.

**Case presentation:**

A 50-year-old Han Chinese man presented to the hospital with decreased vision in his left eye. Examination revealed mild vitritis, papilledema, retinal hemorrhages, and peripheral vascular sheathing in the left eye, raising suspicion of retinal vasculitis. In the following hours, his condition worsened dramatically, with the development of hypopyon and severe vitritis obscuring the visualization of the fundus, suggesting endophthalmitis. He subsequently underwent urgent anterior chamber irrigation, vitreous tap, and intravitreal injection. As the symptoms did not improve, a vitrectomy was performed. The culture results identified the presence of *Abiotrophia defectiva*. Following prompt and effective treatment, the patient’s visual acuity showed improvement.

**Conclusion:**

This report delineates a rare case of endophthalmitis caused by *Abiotrophia defectiva* initially presenting as retinal vasculitis. It emphasizes the need for prompt recognition and treatment of atypical pathogens in postoperative ocular infections to enhance visual outcomes.

## Background

Endophthalmitis is one of the most serious complications following glaucoma filtering surgery, with an incidence of 0.1–0.2% [1, 2]. The most common pathogens include *Streptococcus*, *Enterococcus*, and *Haemophilus influenza* [2, 3], while endophthalmitis caused by *Abiotrophia defectiva* is extremely rare. We present a case of endophthalmitis following trabeculectomy caused by *Abiotrophia defectiva*, where retinal vasculitis was first observed in the early stages of the disease. It was previously compared with other cases observed during vitrectomy. With prompt treatment, the patient achieved a better visual outcome.

## Case presentation

A 50-year-old Han Chinese man presented with mild congestion and decreased vision in his left eye for 12 hours on 19 December 2021. He underwent an uneventful trabeculectomy for open-angle glaucoma in his left eye 2 months earlier and received a subconjunctival injection of 5-fluorouracil (5-FU; 0.3 ml/7.5 mg) to prevent conjunctival filtering bleb scarring 1 day prior. His previous medical history included an appendectomy 18 years ago and a cold 2 weeks ago.

**Fig. 1:**
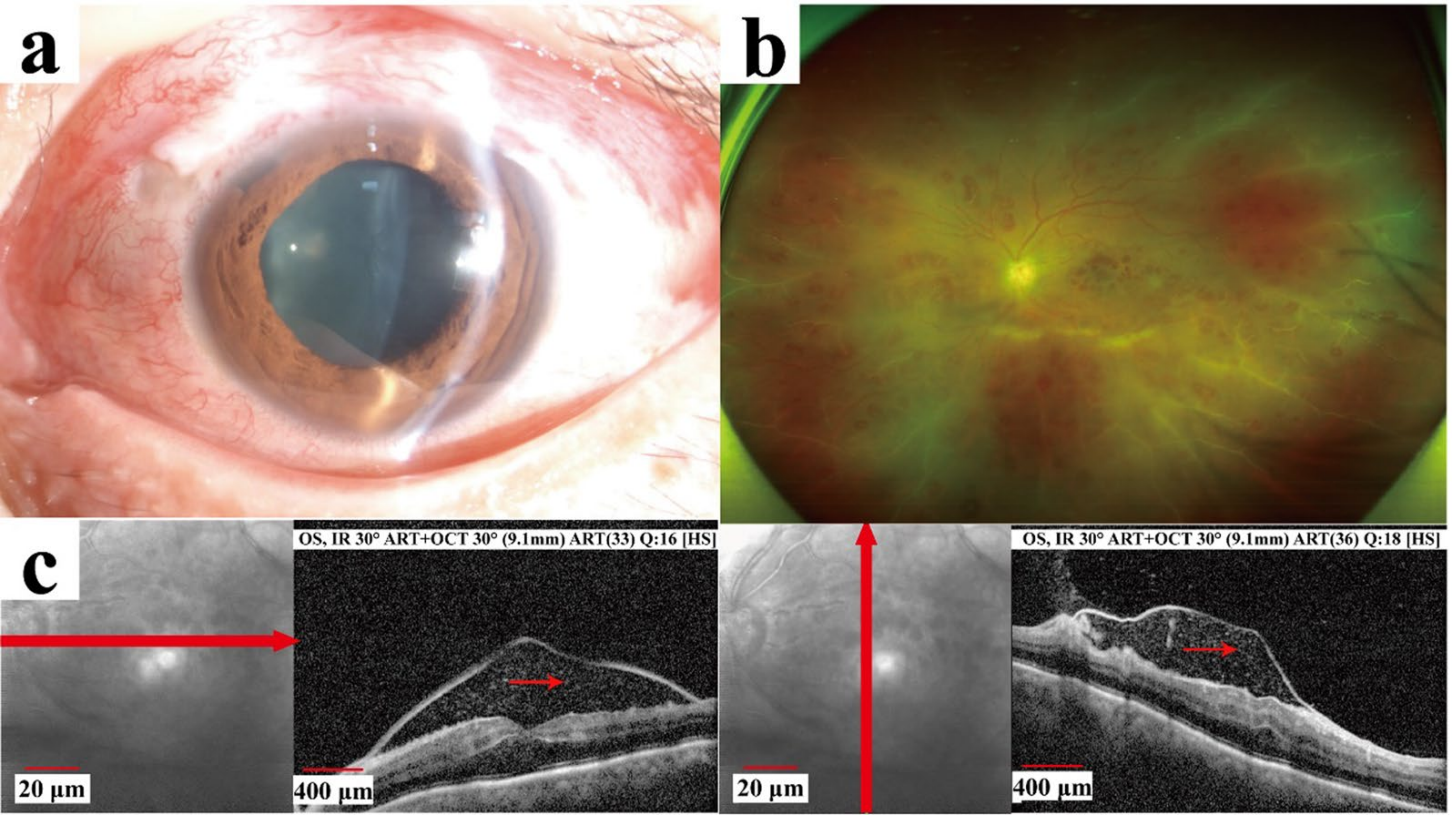
Images were obtained in the outpatient clinic. **a**: The anterior segment showed conjunctival hyperemia, a flat filtering bleb, and a clear cornea with mild anterior chamber cells. **b**: The fudus showed papilledema, retinal hemorrhages, and peripheral vascular sheathing. **c:** Optical coherence tomography revealed a mass of low reflective lesions in the macular area (red arrow showing low reflective lesions)

The visual acuity was 20/160 in the left eye, with an intraocular pressure (IOP) of 30 mm Hg. Slit lamp examination revealed conjunctival hyperemia, a flat filtering bleb, a clear cornea with mild anterior chamber cells, and peripheral iridectomy in the superior nasal quadrant (Fig. 1a). There was mild vitritis, papilledema, retinal hemorrhages, and peripheral vascular sheathing. Optical coherence tomography (OCT) revealed a mass of low reflective lesions in the macular area (Fig. 1b c). The visual acuity in the right eye was 20/25, with an IOP of 17.8 mm Hg after using three eye drops (carteolol twice a day, brimonidine tartrate twice a day, and latanoprost once a day). The anterior segment was normal, and the fundus showed a pale optic disk with a cup-disc ratio of 0.8.

After a routine blood test, and corona virus disease-2019 (COVID-19), the patient was admitted to the hospital after 6 hours, with the suspicion of endophthalmitis in his left eye.

**Fig. 2:**
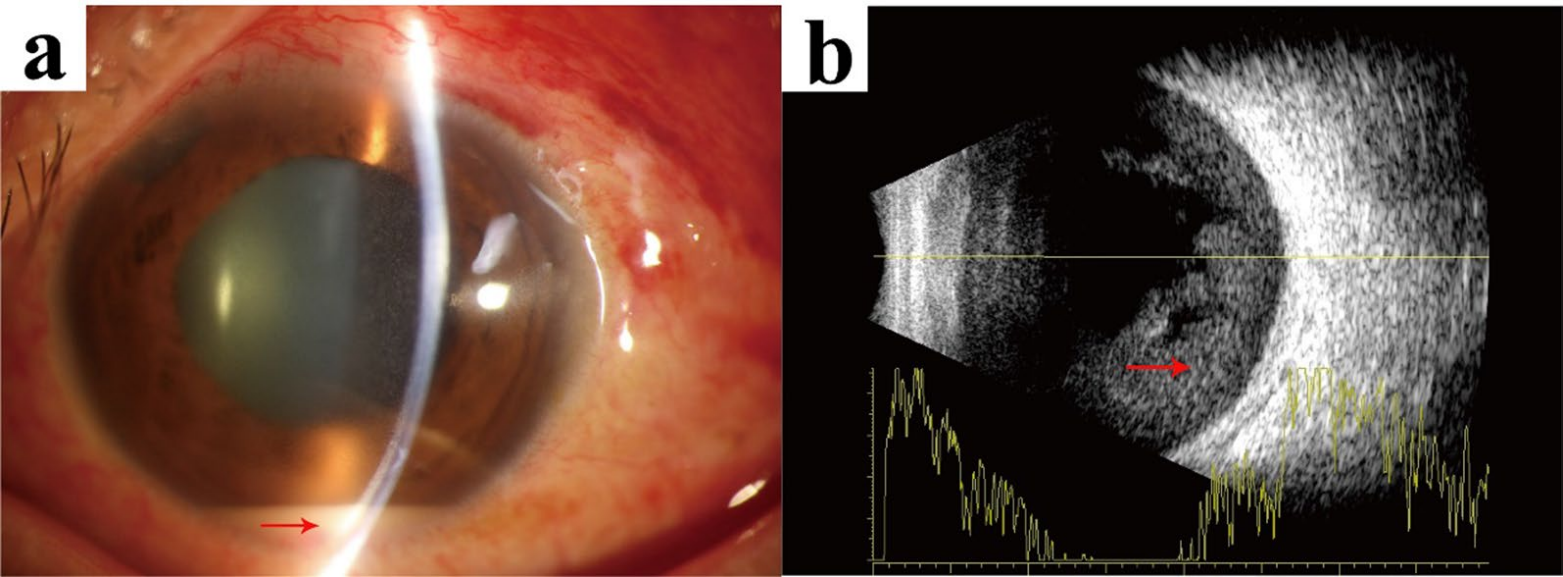
Images were obtained following the patient’s hospitalization. **a**: Slit lamp examination revealed a 1.5-mm hypopyon (red arrow showing hypopyon). **b**: A brightness scan ultrasound confirmed dense vitreous opacities (red arrow showing vitreous opacity)

After being hospitalized, the patient’s condition worsened. The visual acuity dropped to counting fingers at 30 cm, and a significant presence of anterior chamber cells with a 1.5-mm hypopyon was observed (Fig. 2a). The vitritis worsened, obstructing the visualization of the fundus. A brightness (B)-scan ultrasound confirmed dense vitreous opacities (Fig. 2b). Subsequently, 3 hours after being hospitalized, he was promptly transferred to the operating theater for the collection of aqueous humor and vitreous samples for microbiological analysis. He then underwent anterior chamber irrigation and received an intravitreal injection of vancomycin (1 mg/0.1 mL) on 19 December 2021.

**Fig. 3:**
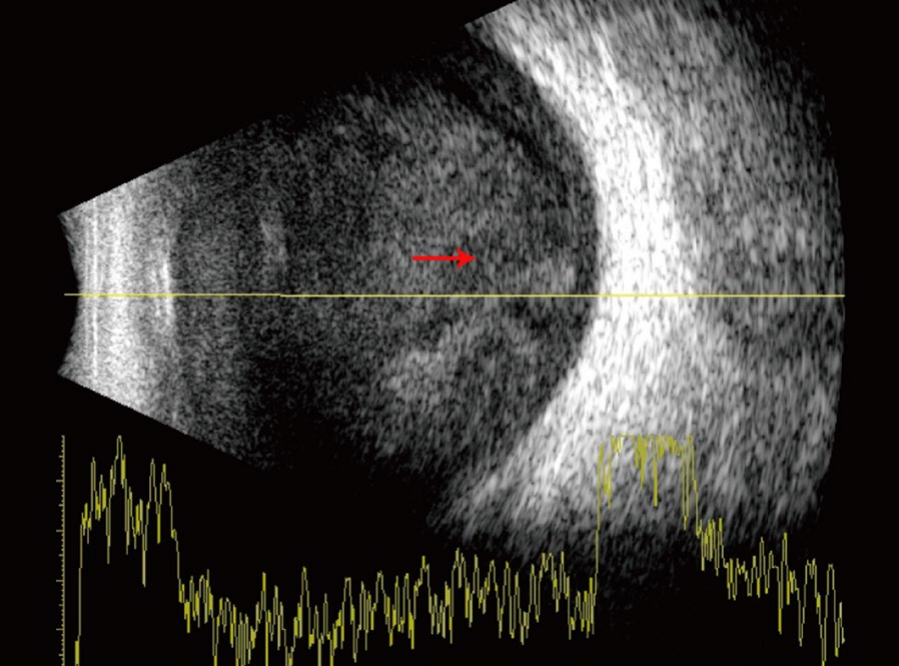
The disease aggravated progressively. Brightness scan ultrasound showed more dense vitreous debris (red arrow showing aggravated vitreous opacity) compared with Fig. 2b

**Fig. 4:**
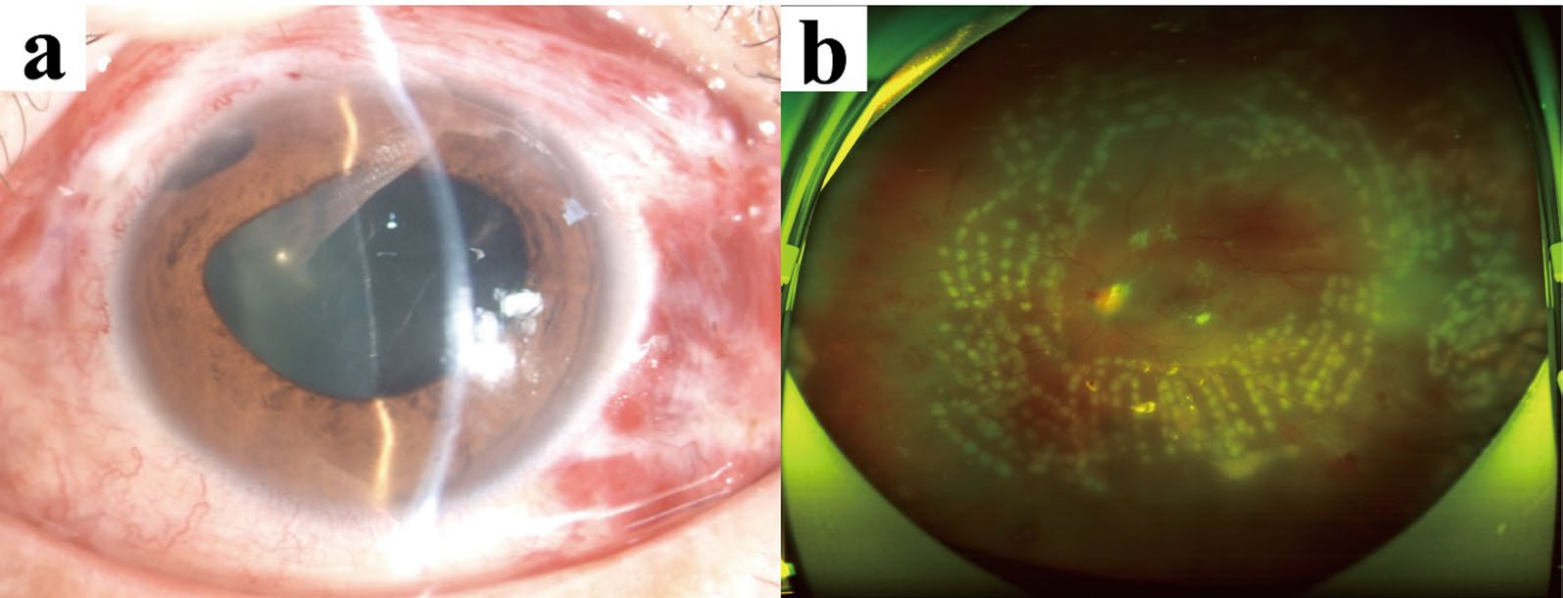
Images were obtained following vitrectomy. **a**: The cornea appeared clear, and no hypopyon was present in the deep anterior chamber. **b**: Blood flow was restored in the peripheral retinal vessels, accompanied by numerous laser spots in the mid-peripheral retina and occasional dot hemorrhages

However, the patient’s condition was not improved, and the disease worsened progressively on 20 December 2021. We could not definitively rule out a viral infection. The patient was administered ganciclovir at a dose of 250 mg twice daily via intravenous injection. The hypopyon increased in size, and the B-scan ultrasound continued to reveal dense vitreous debris (Fig. 3). As a result, a pars plana vitrectomy was promptly performed, followed by an intravitreal injection of vancomycin on 20 December 2021. On the first day post-vitrectomy, the patient’s visual acuity improved to 20/400, and the hypopyon disappeared (Fig. 4a). The peripheral retinal vessels showed restoration, with numerous laser spots in the mid-peripheral retina and sporadic dot hemorrhages (Fig. 4b). Analysis of the aqueous humor on 21 December 2021 did not detect the nucleic acids of cytomegalovirus (CMV), herpes simplex virus (HSV), Epstein–Barr virus (EBV), or varicella zoster virus (VZV). The negative results from the aqueous humor viral tests excluded the possibility of endophthalmitis due to a viral infection. Consequently, antiviral therapy was discontinued. On the third day post-vitrectomy, on 23 December 2021, *Abiotrophia defectiva* was grown using microbiological techniques. According to the antibiogram, the patient was given intravenous drip of vancomycin of 1000 mg every 8 hours for 3 days, followed by intravenous drip of vancomycin of 500 mg every 12 hours for 3 days. Tobramycin, fusidic acid, prednisolone acetate, and compound tropicamide were used topically. On the 9th day post-vitrectomy, the patient’s visual acuity improved to 20/125, with an IOP of 13 mm Hg, achieved without the use of antiglaucoma medications. The fundus examination confirmed the resolution of macular edema and the presence of laser spots. After receiving a peribulbar injection of vancomycin (25 mg/0.5 mL), the patient was discharged on 29 December 2021, with a regimen of topical tobramycin, to be administered three times daily.

The patient was closely monitored following the surgery. Subsequently, 2 months later, he underwent a combined procedure of silicone oil removal, phacoemulsification, and intraocular lens implantation. At the 2-year follow-up, the patient’s condition remained stable without any recurrence of infection. The best corrected visual acuity was 20/20 in the right eye and 20/32 in the left eye, with IOP of 10.8 mm Hg and 14.8 mm Hg, respectively.

## Discussion

*Abiotrophia defectiva*, classified as nutritionally variant streptococci, is part of the normal flora of the oral cavity, gut, and urogenital tracts of humans [4]. Additionally, it can also be acquired from the ocular surface, rarely leading to conjunctivitis and keratitis [5]. Endocarditis is most frequently reported as being caused by *Abiotrophia defectiva* [6, 7]. However, until the time of submission, there have been very few reports of *Abiotrophia defectiva* causing endophthalmitis in literature, including five cases following conventional cataract surgery [4, 8–10], three cases of keratopathy [5, 11, 12], two cases after trabeculectomy [13, 14], and one case after intravitreal injection of dexamethasone [15]. All the cases of endophthalmitis caused by *Abiotrophia defectiva* are summarized in Table 1. The rapid progression of endophthalmitis caused by *Abiotrophia defectiva* results in severe vitritis, which hinders the view of the fundus. In contrast to all published cases of *Abiotrophia defectiva* endophthalmitis, we were the first to observe retinal vasculitis in the very early stages of this disease (within 12 hours).

**Table 1:** Reported cases of endophthalmitis due to *Abiotrophia defectiva*

Survey	References		Age, years	Sex	Initial visual acuity	Initial eye disease	Complication	Intraocular antimicrobial therapy	Vitrectomy	Systemic therapy	Outcome
Cataract surgery	[4]		65	Female	Light perception	Cataract	No	Vancomycin + ceftazidime	Yes	Oral ciprofloxacin and prednisolone	6/9.5 with pinhole after 9 weeks
	[8]	Case 1	83	Male	Hand movement	Cataract	No	Vancomycin + amikacin	Yes	Oral steroids	Not reported
		Case 2	80	Female	Hand movement	Cataract	No	Vancomycin + ceftazidime	Yes	Ciprofloxacin	Not reported
	[9]		79	Female	Light perception	Cataract	Retinal disease; infiltrative keratitis	Vancomycin + cefotaxime	Yes	Oral imipenem and ciprofloxacin	20/400 after 5 months
	[10]		72	Male	Reduced	Cataract	No	Vancomycin + amikacin	No	Intravenous vancomycin	Not reported
Keratopathy	[5]		83	Female	Counting fingers	Pseudophakic bullous keratopathy	No	Vancomycin + ciprofloxacin	No	No	20/60 after 1 year
	[11]		78	Male	Reduced	Pseudophakic bullous keratopathy	No	Vancomycin, moxifloxacin, and natamycin	No	Oral ciprofloxacin	20/80 after 3 months
	[12]		77	Male	Counting fingers	Bilateral lattice stromal corneal dystrophy	The secondary graft failure	Topical cefazolin + ciprofloxacin	No	No	20/400 after 3 weeks
Trabeculectomy	[13]		74	Female	Light perception	Primary open-angle glaucoma	No	Vancomycin + ceftazidime	No	Oral ciprofloxacin	6/18 (final visual acuity)
	[14]		75	Female	Hand movement	Primary open-angle glaucoma	Intraretinal hemorrhages	Vancomycin + ceftazidime	Yes	Oral levofloxacin	20/40 after 2 months
Intravitreal injection of dexamethasone	[15]		70	Female	Counting fingers	Branch vein occlusion, macular edema	Significant retinal vascular attenuation with macular atrophy	Vancomycin + ceftazidime	Yes	No	Hand movement

Improper handling of samples, transportation delays, and specific growth requirements may have contributed to negative findings and the scarcity of reports. In our case, we observed that positive vitreous cultures were successfully obtained on the 3rd day following vitrectomy. This timeline contrasts with the findings reported by Debarshi, where positive cultures were noted on the 2nd day, and with Ming-Han’s report, which indicated positive results on the 17th day post-procedure [13, 14]. *Abiotrophia defectiva* is categorized under the group of nutritionally variant streptococci. These bacteria are distinguished by their unique growth requirements, specifically their need for L-cysteine supplementation to thrive in conventional culture media. Furthermore, L-alanine plays an indispensable role in the biosynthesis of peptidoglycan, which is a fundamental structural element of bacterial cell walls. Therefore, it is speculated that the presence of L-cysteine and L-alanine in the culture medium of vitreous specimens may facilitate the growth of *Abiotrophia defectiva* [14].

Retinal vasculitis is one of the early signs of exogenous endophthalmitis, often caused by the bacterial exotoxins and subsequent immune responses [16, 17]. In the early stage of our case, fundus photography revealed retinal hemorrhages and peripheral vascular sheathing. To the best of our knowledge, this is the first case report describing these findings in *Abiotrophia defectiva* endophthalmitis.

Notably, *Abiotrophia defectiva* is a rare cause of endophthalmitis. The rapid progression of fundus lesions may be correlated with the binding ability and virulence of *Abiotrophia defectiva*. However, *Abiotrophia defectiva* exhibits a strong affinity for fibronectin and produces significant amounts of extracellular polysaccharides, which can adhere to fibronectin at the junction between the vitreous and retinal nerve fibers. Moreover, the deposition of antibody–antigen complexes in vessel walls may lead to vascular damage and an inflammatory response.

Subconjunctival injection of 5-fluorouracil is a common clinical practice to prevent postoperative scarring of filtering blebs. The incidence of filtering bleb-associated endophthalmitis is higher with adjunctive 5-fluorouracil therapy during trabeculectomy compared with when it is not used [18, 19]. Bacterial migration in the conjunctiva is the primary cause [20].

We quickly ruled out viral retinitis for the following reasons. Firstly, viral retinitis is characterized by yellow–white patchy necrosis foci, which were not present in our case. Secondly, we conducted a virological test on the aqueous humor before the initial vitreous tap, without any antiviral drugs, and the test did not detect viral nucleic acid of CMV, HSV, EBV, or VZV.

In the case of endophthalmitis, the key components of the treatment plan include an initial vitreous tap and intravitreal injection of antibiotics, with current recommendations favoring vancomycin combined with ceftazidime. In published case reports of *Abiotrophia defectiva* endophthalmitis, Namdari, Mustafi, and Yousef *et al*. found that patients who received prompt vitrectomy had significantly better visual outcomes [8, 14, 15]. Han and Madalina *et al*. reported that timely vitreous taps and multiple vitreous injections resulted in good visual outcomes for patients [4, 13]. Our patient underwent urgent anterior chamber irrigation, vitreous tap, and intravitreal injection; however, the vitritis continued to progress. Consequently, we performed a vitrectomy the day after the intravitreal injection, and with targeted antimicrobial therapy, the patient achieved an improved visual outcome. Owing to the limited number of case reports, further research on *Abiotrophia defectiva* endophthalmitis after following glaucoma surgery is needed.

## Conclusion

In clinical practice, accurately identifying the pathogen responsible for endophthalmitis is crucial for effective and targeted treatment. Retinal vasculitis can be an early sign of *Abiotrophia defectiva* endophthalmitis. Timely vitreous tap, vitrectomy, and effective antimicrobial therapy are essential for achieving good visual outcome in patients.

## Data Availability

All data in this study are available from the corresponding author upon reasonable request.

## Abbreviations

IOP: Intraocular pressure
OCT: Optical coherence tomography
COVID-19: Corona virus disease-2019
CMV: Cytomegalovirus
HSV: Herpes simplex virus
EBV: Epstein–Barr virus
VZV: Varicella zoster virus
